# Sorafenib treatment for papillary thyroid carcinoma with diffuse lung metastases in a child with autism spectrum disorder: a case report

**DOI:** 10.1186/s12885-017-3782-7

**Published:** 2017-11-21

**Authors:** Yousuke Higuchi, Takayuki Motoki, Hisashi Ishida, Kiichiro Kanamitsu, Kana Washio, Takanori Oyama, Takuo Noda, Yasuko Tsurumaru, Ayumi Okada, Hirokazu Tsukahara, Akira Shimada

**Affiliations:** 1 Department of Pediatrics, Okayama University Hospital, 2-5-1 Shikata-cho, Kita-ku, Okayama, 700-8558 Japan; 2 Department of General Thoracic Surgery and Breast and Endocrine Surgery, Okayama University Hospital, 2-5-1 Shikata-cho, Kita-ku, Okayama, 700-8558 Japan; 3 Department of Pediatric Surgery, Okayama University Hospital, 2-5-1 Shikata-cho, Kita-ku, Okayama, 700-8558 Japan; 4 Department of Pediatrics, Okayama University Graduate School of Medicine, Dentistry and Pharmaceutical Sciences, 2-5-1 Shikata-cho, Kita-ku, Okayama, 700-8558 Japan

**Keywords:** Sorafenib, Pediatrics, Papillary thyroid carcinoma, Lung metastases, Autism spectrum disorder

## Abstract

**Background:**

Pediatric papillary thyroid carcinoma frequently presents with lymph node involvement and distant metastases. Sorafenib, an oral multikinase inhibitor, has been used to treat radioactive iodine (RAI) therapy-refractory thyroid carcinoma in adults; however, pediatric experience is limited. Medical procedures and hospitalization for children with autism spectrum disorder may be challenging.

**Case presentation:**

An 11-year-old boy with autism spectrum disorder and moderate intellectual impairment presented with dyspnea on exertion with thyroid carcinoma and diffuses lung metastases. Total thyroidectomy and adjuvant RAI therapy is the standard treatment; however, the latter therapy was impractical because of his respiratory status and challenging behaviors. He was therefore started on sorafenib 200 mg/day (150 mg/m^2^/day) and this dosage was increased to 400 mg/day (300 mg/m^2^/day). The adverse effects were mild and tolerable. After administration of medication, his dyspnea improved and surgery was performed. We attempted to administer RAI therapy after surgery; however, we abandoned it because he had difficulty taking care of himself according to isolation room rules. Thyrotropin suppression therapy was therefore started and sorafenib treatment (400 mg/day) resumed. Follow-up imaging showed regression of pulmonary metastases. The metastases have remained stable for over 24 months on continuous sorafenib treatment without serious adverse events.

**Conclusion:**

We inevitably used sorafenib as an alternative to standard therapy because of the patient’s specific circumstances. Individualized strategies for pediatric cancer patients with autism spectrum disorder are needed.

## Background

Thyroid carcinoma is rare in the pediatric population and its incidence in the United States in 2010–2014 was 0.4 per 100,000 in 10–14-year-old male individuals [1]. Papillary thyroid carcinoma (PTC) is the most common thyroid malignancy. Although PTC is usually indolent, pediatric patients with PTC frequently present with distant metastases [2]. Even though cervical lymph node involvement and pulmonary metastases are common, the prognosis is excellent [2, 3]. This positive prognosis is considered a result of good response to radioactive iodine (RAI) therapy in pediatric patients with PTC and pulmonary metastases [4, 5].

Aberrant activation of mitogen-activated protein kinase signaling pathways is critical for thyroid carcinoma [6]. *BRAF* (especially *V600E*), *RAS* point mutations and *RET/PTC* rearrangements are common genetic abnormalities in PTC. Sorafenib, an oral multikinase inhibitor that inhibits BRAF, CRAF, VEGF receptors 1 to 3, platelet-derived growth factor receptors, and RET, is approved by the United States Food and Drug Administration for adults with RAI therapy refractory well-differentiated thyroid carcinoma [7, 8]. However, the safety and effectiveness of sorafenib in pediatric patients have not been established. In Japan, sorafenib was approved for unresectable thyroid cancer without age regulation, but there was no experience of using sorafenib in pediatric patients.

Autism spectrum disorder (ASD) is neurodevelopmental impairments of communication, socialization and repetitive behaviors, and frequently co-occur with intellectual disability [9]. The prevalence of ASD is approximately 1 in 68 8-years-old children [10]. Children with ASD tend to engage in their routines and are resistant to change. Therefore, medical procedures and hospitalization for children with ASD may provoke challenging behaviors [11].

Here we report a child with ASD who developed PTC with diffuse lung metastases and was treated with sorafenib.

## Case presentation

**Fig. 1:**
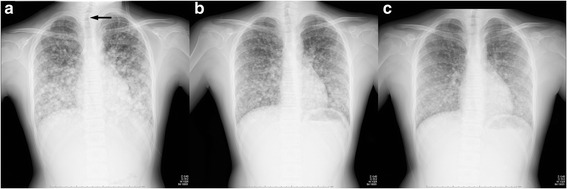
Chest radiographs. **a** Chest radiograph showing innumerable lung nodules in a miliary pattern. The trachea is deviated to the right (*arrow*). **b** In 2 months after receiving sorafenib and before surgery showing the relief of deviated trachea. **c** In 2 month after surgery and resume sorafenib showing improvement in the lung nodules

**Fig. 2:**
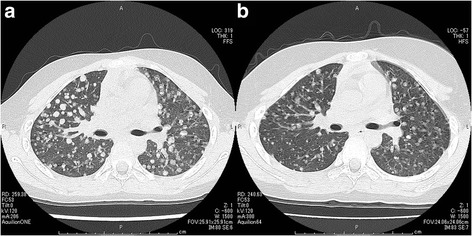
Radiographic changes in lung metastases. **a** Chest computed tomography scan showing innumerable metastases. **b** In 2 month after surgery and resume sorafenib showing regression of lung metastases

An 11-year-old boy with ASD and moderate intellectual disability was taken to regional hospital because of dyspnea on exertion. The breath sounds were diminished, percutaneous oxygen saturation (SpO_2_) was 89% and 97% on 5 L/min of oxygen. A chest radiograph showed diffuse pulmonary nodules and tracheal deviation to the right (Fig. 1a). A computed tomography scan of the chest and neck revealed innumerable small nodules throughout both lungs (Fig. 2a), a 2-cm nodule in the left thyroid lobe, and enlarged cervical lymph nodes. He was referred to our department for further examination and treatment. His parents were not consanguineous and there was no history of radiation exposure or family history of cancer. Laboratory data showed markedly increased serum thyroglobulin (Tg) concentrations (2206 μg/L, normal range: 0.0–32.7 μg/L) with negative Tg autoantibody (< 6.1 kU/L, normal range: < 13.6 kU/L). Fine-needle aspiration cytology of the thyroid nodule confirmed malignancy.

Total thyroidectomy and adjuvant RAI therapy is the standard treatment for such patients; however, these therapies were considered impracticable immediately because of his respiratory status and challenging behaviors (he frequently pulled his oxygen mask off and ran out of the room; therefore, he needed an attendant full time). Even though the safety and effectiveness of sorafenib in children have not been established, our medical team including pediatricians, pediatric surgeons, endocrine surgeons and radiologists discussed potential risks and benefits with his family. After obtaining written informed consent, we decided to prescribe sorafenib. Treatment was started at 200 mg (150 mg/m^2^/day) in two divided doses per day (BID) and increased to 400 mg (300 mg/m^2^/day) BID because his Tg concentration increased to 3600 μg/L. The dose was decided based on a Children’s Oncology Group phase 1 study of sorafenib in children with refractory solid tumors and leukemia [12]. We performed physical examination including blood pressure measurement and weekly laboratory examination including serum proteins, bilirubin, aminotransferase, amylase, creatinine, electrolytes, and complete blood counts to evaluate the toxicity of sorafenib. An ordinary moisturizing cream was provided for skin care to prevent a hand–foot skin reaction [13]. The adverse effects of increased aminotransferase concentration (Common Terminology Criteria for Adverse Events [CTCAE] version 4.0, Grade 1), mild diarrhea (CTCAE Grade 1), and hand rash (CTCAE Grade 1) occurred [14]. After 2 months of receiving sorafenib, his dyspnea on exertion had improved and chest radiograph showed relief of tracheal deviation (Fig. 1b). Meanwhile, Tg concentrations had remained within the range of 1500–2000 μg/L. Then, total thyroidectomy and lymph node dissection were performed 1 week after cessation of sorafenib. The left lobe was excised as completely as possible, with tumor invasion to trachea preventing total excision. The left swollen deep lateral cervical lymph nodes were dissected but superior internal jugular lymph nodes were left in situ because of adhesions to the vagus nerve. His postoperative course was uneventful and there were no surgery-related complications. Histopathologic examination of the resected specimen revealed PTC of mixed unencapsulated follicular variant pattern, Stage T4_a_, N1_b_, M1 (American Joint Committee on Cancer Staging Manual 7th edition) with positive surgical margins [15].

An RAI whole body scan showed uptake in the thyroid remnant and lungs. We attempted to administer RAI therapy; however, abandoned it eventually because he had difficulty taking care of himself according to isolation room rules (e.g. drink 1.5 L of water per day, change clothes, and excrete in a designated area) because of his inflexible character and intellectual disability (Tanaka–Binet Intelligence quotient test showed his mental age was 5 years). Thyrotropin suppression therapy was therefore started and sorafenib treatment (400 mg BID) resumed. Two months after surgery, he was able to ambulate with oxygen at 1 L/min. Follow-up imaging revealed regression of pulmonary metastases (Fig. 1c and 2b). The metastases have remained stable for over 24 months on consecutive sorafenib treatment (400 mg BID) without serious adverse event including growth plate widening at his wrists and knees (his bone age was 14 years and consistent with chronological age). Tg concentrations declined to around 400 μg/L and have also remained stable for over 24 months. After receiving informed consent from the patient’s parents and with the approval of the Ethics Committee of Okayama University Hospital, we conducted genetic analysis. No common mutations seen in PTC were identified in *BRAF, HRAS* or *KRAS*.

## Discussion and conclusions

The American Thyroid Association guidelines Task Force on Pediatric Thyroid Cancer proposes the following management for patients with known distant metastases: monitoring of Tg on thyrotropin suppression therapy and performing an RAI whole body scan if Tg concentrations increase [16]. If RAI uptake is confirmed, the patient is treated with RAI therapy. Most children with pulmonary metastases have micronodular disease, which typically has excellent RAI uptake. Therefore, we thought that RAI therapy was needed and attempted to administer it after surgery; however, we were not sure if the patient could receive RAI therapy at that time because of his challenging behaviors. We considered that the significance of total thyroidectomy alone was limited. Therefore, we administered sorafenib expecting of his lung metastases that would allow enough time to train him to receive RAI therapy. Although our nurses, paramedical staff, and his family worked diligently over an extended period time to train him, he remained unable to obey isolation room rules. This, unfortunately, prevented us from administering RAI therapy. Children with ASD have more anxiety and behavioral conduct problems than children without ASD, and challenging behaviors including non-compliance complicate the treatment [13]. Developing individual approach is important for the management of children with ASD presenting with fatal disease such as cancer.

There are several subtypes of PTC; the follicular variant of PTC has a follicular architectural pattern and nuclear features similar to those of classical PTC. Patients with follicular variant of PTC and extrathyroidal extension of their tumor or distant metastases have a higher disease-specific mortality than those with classical PTC [17]. Our patient also had infiltrative tumor and miliary pulmonary metastases. We identified no somatic mutations in *BRAF, HRAS,* or *KRAS* in our patient. However, *NRAS* and chromosomal rearrangements were not included in our sequence analysis. Some recent reports have used next-generation sequencing to investigate both conventional and rare mutations and chromosomal rearrangements in pediatric patients with PTC [18, 19].

Sorafenib improves progression free survival in patients with well-differentiated thyroid carcinoma [20, 21]. Previous studies of sorafenib in pediatric patients with PTC have reported marked reduction in pulmonary metastases [22, 23]. Rash, hand–foot skin reaction, gastrointestinal symptoms, fatigue, and hypertension are commonly observed adverse effects of sorafenib [13]. Our patient showed clinical and radiographic improvement after administration of sorafenib and the adverse effects were mild and tolerable. Furthermore, sorafenib can be administered in an outpatient setting and is easily managed. In fact, the patient can take oral medication by collaborating with his family. However, thyroid remnant and metastases remain as yet and sorafenib is expensive drug. We would conduct RAI therapy if the patient were to take care of himself. The Children’s Oncology Group recently described a phase 2 study of sorafenib in refractory solid tumors; unfortunately, no patients with PTC were enrolled in this study [24]. Furthermore, growth plate abnormalities were observed in young animals with tyrosine kinase inhibitors including sorafenib, and growth plate widening were reported in pediatric cancer patients undergoing phase 1 studies of tyrosine kinase inhibitors [25]. Unfortunately, we did not evaluate his growth plate before the treatment, but he showed no unequivocal growth plate widening after over 24 months of receiving sorafenib. Further studies are required to evaluate the effectiveness and long-term safety of this molecular target inhibitor in pediatric patients with PTC.

In conclusion, we report the case of a PTC with diffuse pulmonary metastases in a pediatric patient with ASD. We inevitably used sorafenib as an alternative to standard therapy because of the patient’s specific circumstances. Individualized strategies for pediatric cancer patients with ASD are needed.

## Abbreviations

ASD: Autism spectrum disorder
BID: Two divided doses per day
CTCAE: Common Terminology criteria for Adverse Events
PTC: Papillary thyroid carcinoma
RAI: Radioactive iodine
SpO2: Percutaneous oxygen saturation
Tg: Thyroglobulin
